# Case-Based Virtual Escape Rooms: Using Feedback to Change Online Platforms

**DOI:** 10.7759/cureus.49805

**Published:** 2023-12-01

**Authors:** Kaitlyn Boggs, Manu Madhok, Tania Ahluwalia

**Affiliations:** 1 Pediatric Emergency Medicine, Medical University of South Carolina, Charleston, USA; 2 Pediatric Emergency Medicine, Children's Minnesota, Minneapolis, USA; 3 Pediatric Emergency Medicine, Children's National Medical Center, Washington, DC, USA

**Keywords:** toxicology, pediatric emergency medicine, feedback, case-based teaching, virtual escape room

## Abstract

Introduction

Virtual escape rooms (VERs) have provided education in healthcare settings. VERs were developed to provide medical education related to pediatric toxicology. This study explores simulation technologies, specifically portals to create and host VERs, including Google Sites, a website-building platform, and Articulate 360, an e-learning platform. The design objective was to create an engaging educational tool using a VER on two pediatric toxicology scenarios.

Methods

Case-based VERs were developed on Google Sites and Articulate 360. The cases focused on organophosphate toxicity and acute iron toxicity. Google Sites technology was used to build the organophosphate toxicity case, which was implemented and piloted with emergency medicine (EM) trainees in India and workshop participants at the International Pediatric Simulation Symposia and Workshops (IPSSW) in 2022. The iron toxicity case was developed using Articulate 360 and piloted at IPSSW in 2023. Feedback was collected as a survey from participants. Questions focused on using VERs as an engaging educational model, benefits, areas for improvement, and future participation in VERs. Following the study, a focus group meeting was held with facilitators and developers and subsequently analyzed.

Results

Evaluations from participants and a focus group provided data demonstrating both platforms' utility. Participants completed surveys after each VER. Overall, 84.2% of respondents (n=60) from EM training programs in India, 90.9% of respondents (n=11) from IPSSW in 2022, and 100% of respondents (n=23) from IPSSW in 2023 agreed or strongly agreed that this was an engaging education model.

Conclusion

Different platforms may be used to develop engaging VERs for gamification in education. This study found that VERs based on pediatric toxicology scenarios created on Google Sites and Articulate 360 are engaging educational tools for distance learning. Simulation technologies have benefits and disadvantages for Google Sites and Articulate 360. Simulation developers and educators should consider time, funding, technological needs, and participant feedback when deciding which portal to choose when building a VER.

## Introduction

Pediatric emergency medicine (PEM) training is crucial to provide physicians with the knowledge and skills to manage acutely ill children, including those with toxic ingestions. Virtual escape rooms (VERs) are adaptations of in-person escape rooms, which use online interactive environments where participants aim to solve puzzles within a designated timeframe [1]. VERs have been proven effective in healthcare education [1-7]. In this project, VERs were developed for medical education on pediatric ingestions. This study explores simulation technologies, specifically portals to create and host VERs, including Google Sites and Articulate 360. Google Sites is a website-building platform by Google that allows users to create and customize websites while integrating with other Google tools, such as Google Slides, making it easy for users to embed content as needed [8]. Articulate 360 is a subscription-based e-learning platform with various software applications that help facilitate e-learning content [9]. The specific design objective was to create an engaging educational tool in the form of a VER for a global audience.

## Materials and methods

Participants included emergency medicine (EM) trainees in India and participants at the International Pediatric Simulation Symposia and Workshops (IPSSW). The Ronald Reagan Institute of Emergency Medicine (RRIEM) at George Washington University has long-standing partnership programs with institutions across India, including a 3-year Master in Emergency Medicine education and training program [10-11]. Before the COVID-19 pandemic, international EM experts delivered in-person education at partner sites. However, due to travel restrictions during the pandemic, training was expanded to include remote/distance learning, including VERs. The International Pediatric Simulation Society is a pediatric simulation society with members worldwide, with dedicated members providing critical feedback to each other [12].

**Figure 1:**
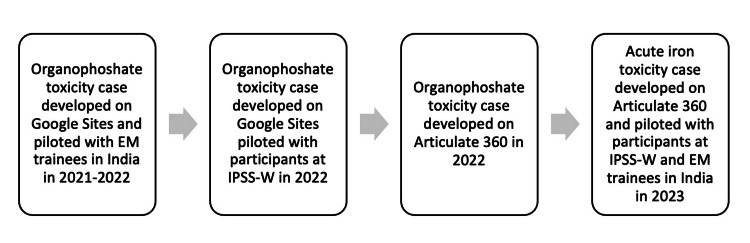
Timeline of virtual escape rooms EM, emergency medicine; IPSSW, International Pediatric Simulation Symposia and Workshops

Case-based VERs were developed using two platforms: Google Sites and Articulate 360. The former was used to build a VER on organophosphate toxicity. Simulation educators (Kaitlyn Boggs, Manu Madhok, and Tania Ahluwalia, who specialize in PEM) developed a facilitator guide with keys, clues, and hints to navigate the case, including patient presentation, physical examination, initial workup, and management. This VER was piloted with EM trainees in India and participants at IPSSW in 2022. Figure 1 depicts a timeline of VERs.

**Figure 2:**
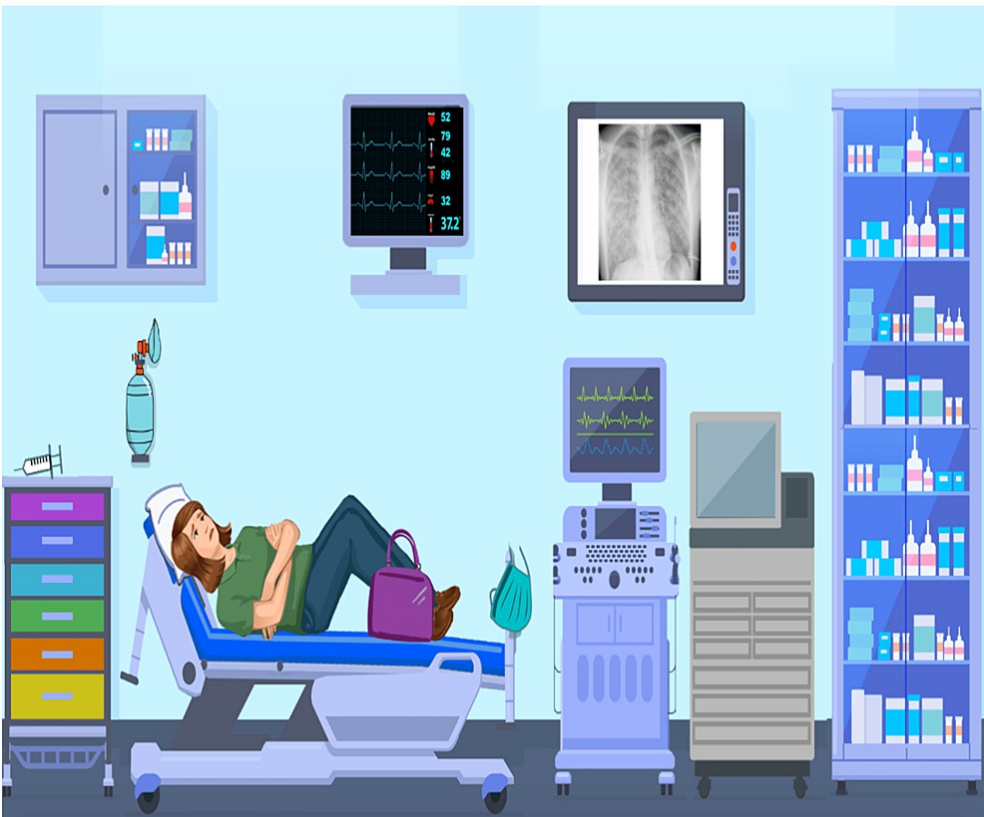
Virtual escape room on organophosphate toxicity developed on Articulate 360 Image credits and permission from Children’s National Hospital Medical Education Department E-Learning Team

Based on the participant’s feedback, the organophosphate case was re-created using the original facilitator guide on Articulate 360 (Figure 2).

**Figure 3:**
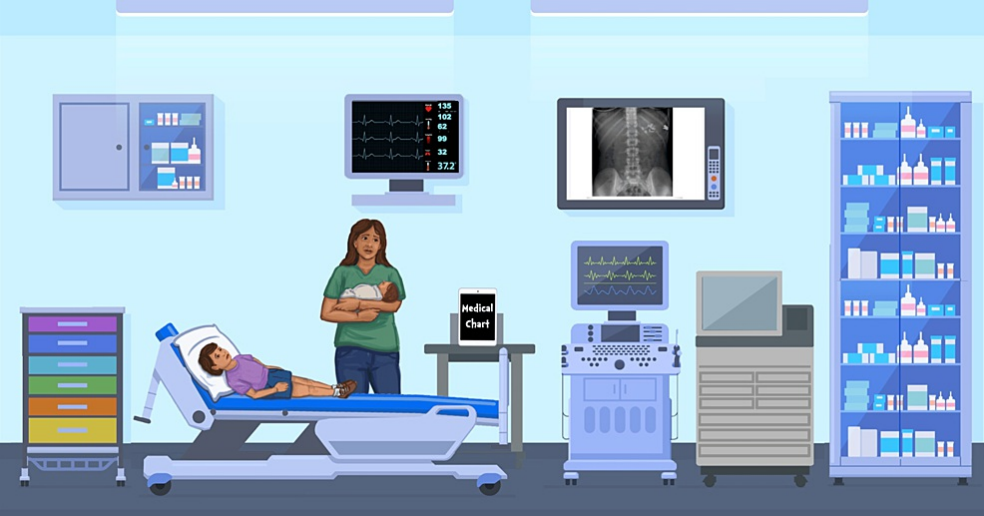
Virtual escape room on acute iron toxicity developed on Articulate 360 Image credits and permission from Children’s National Hospital Medical Education Department E-Learning Team

Then, a different case was developed, focusing on acute iron toxicity using Articulate 360, which was piloted at IPSSW in 2023 (Figure 3).

Participants completed a survey to evaluate the VER. After implementing the VERs and reviewing participant feedback, a focus group meeting was held with facilitators and developers via an online platform, Zoom Video Communications, Inc.

The clues for the Google Sites case were developed using free puzzle websites, whereas the clues for Articulate 360 were constructed within the educational program. Both platforms accommodated puzzles, including word scramble, word search, crossword puzzles, ciphers, quizzes, and matching games. Author K.B. developed the organophosphate case on Google Sites. The Children’s National Hospital Medical Education Department E-Learning Team built VERs on Articulate 360 using a facilitator guide created by the simulation educators.

## Results

**Figure 4:**
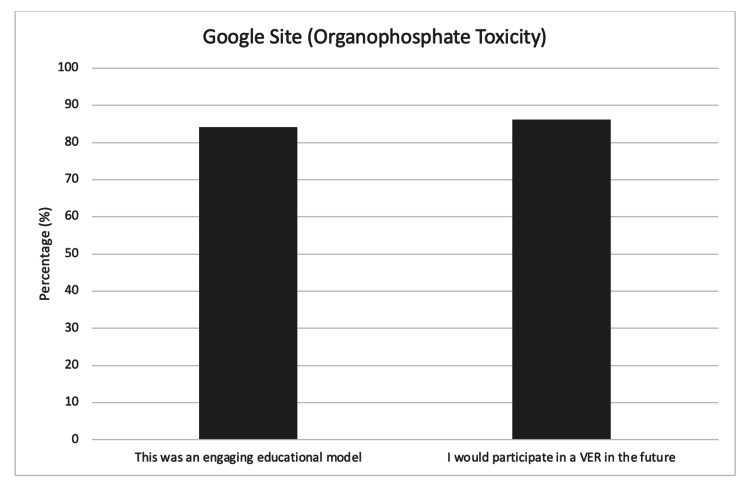
Survey results on the Google Sites VER (organophosphate toxicity) from EM trainees in India EM, emergency medicine; VER, virtual escape room

The organophosphate toxicity case developed on Google Sites was implemented and piloted with EM trainees in India and workshop participants at IPSSW in 2022. All participants from RRIEMs partner programs in India (n=120) and participants at IPSSW (n=14) successfully completed all the puzzles and escaped the VER in the allotted time of 75 minutes. Participants evaluated the VER with a survey. There was a 50% survey response rate from the participants in India (n=60). All respondents were first, second, and third-year EM trainees. Survey questions focused on the usefulness of VERs as an engaging educational model, strengths, areas for improvement, and future participation in VERs. 84.2% of respondents agreed or strongly agreed that this was an engaging educational model (Figure 4).

**Figure 5:**
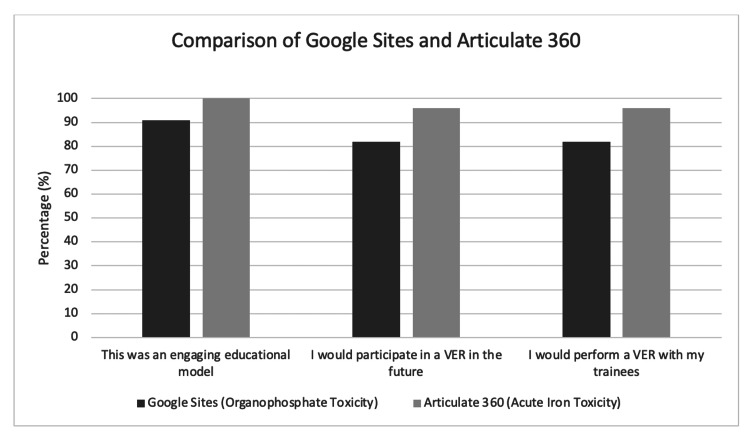
Survey results from IPSSW comparing VERs: Google Sites (organophosphate toxicity) and Articulate 360 (acute iron toxicity) IPSSW, International Pediatric Simulation Symposia and Workshops; VERs, virtual escape rooms

Overall, 86.2% strongly agreed or agreed that they would participate in a VER in the future. Respondents described the VER as “non-traditional,” “different,” “exciting,” and “engaging every second.” One participant shared that “brain-teasing in the medical setting is definitely intriguing.” Another participant stated they enjoyed “the innovated puzzles [that] are not mundane.” At IPSSW in 2022, there was a 78.6% survey response rate (n=11). Respondents were physicians, nurses, and simulation educators, and 90.9% of respondents agreed or strongly agreed that this VER was an engaging educational model (Figure 5).

Overall, 81.8% of respondents agreed or strongly agreed that they would participate in a VER in the future, and 81.8% agreed or strongly agreed that they would perform a VER with their trainees. Participants commented that they enjoyed the “interactivity,” “critical thinking,” and “collaboration.” Respondents from both groups commented on the need for better audiovisual technology, a way to avoid the need for multiple internet tabs, and a way to lock clues to prevent progression through the case.

The organophosphate case was re-created using Articulate 360 after facilitators and developers addressed the identified technological pitfalls. Once the team understood the technical capabilities of Articulate 360, a case on iron toxicity was developed. This case was piloted at IPSSW in 2023. A total of 27 survey respondents included physicians, nurses, and simulation educators. All (100%) of the respondents agreed or strongly agreed that this was an engaging educational model, 96% agreed or strongly agreed that they would participate in a VER in the future, and 96% of respondents agreed or strongly agreed that they would perform a VER with their trainees. Respondents commented that they enjoyed the “virtual format” and “easy accessibility.” They enjoyed “learning about the platform” and the “nice interface.” One respondent commented that they enjoyed this “interactive learning tool that promoted discussion and teamwork.” Another respondent commented that they “found it engaging and a great way to impart new knowledge.” Respondents also commented on the “fun team building” and “team collaboration and interaction.”

Focus groups with facilitators and developers, including physicians and the e-learning team, found benefits and challenges to both technologies. Google Sites is a free, accessible resource but is time-consuming to develop. Google Sites involves using free puzzle creation websites and embedding them into Google Slides to create a VER. On the other hand, Articulate 360 costs more but has more features, including more interactivity, games, and tracking. Articulate 360 was used to address the identified technical pitfalls of Google Sites. The organophosphate toxicity case designed on Google Sites took approximately 120 hours, whereas the organophosphate case constructed on Articulate 360 took about 90 hours. Creating the iron toxicity case on Articulate 360 took about 75 hours. The developers noted that the iron toxicity case took less time after building a patient room and practicing puzzles for the organophosphate case. These estimated hours did not include the time to establish the facilitator guide. Each facilitator guide took approximately 20 hours to develop, and the same facilitator guide was used for the organophosphate case, which was used to build VERs on both Google Sites and Articulate 360. The simulation educators all agreed that it was helpful to collaborate with the e-learning team to lead the technological components to allow them to focus on medical education.

## Discussion

**Figure 6:**
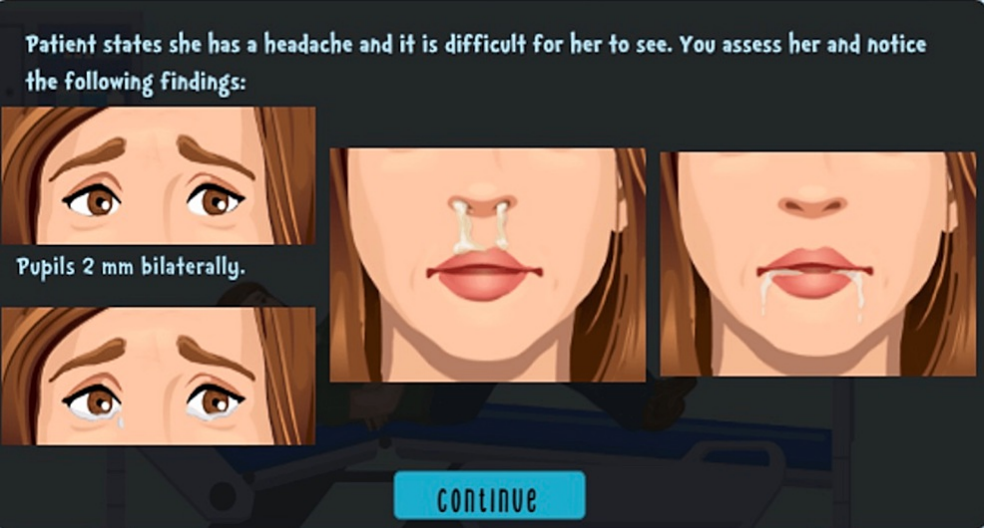
Physical examination findings on Articulate 360 Image credits and permission from Children’s National Hospital Medical Education Department E-Learning Team

**Figure 7:**
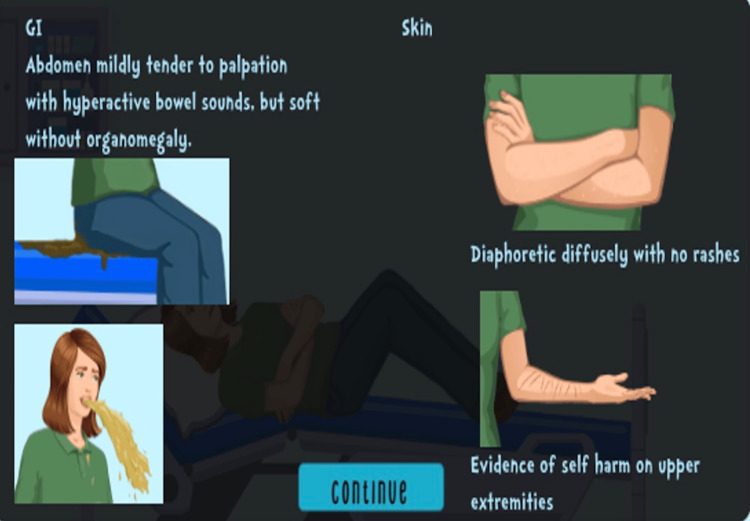
Physical examination findings on Articulate 360 Image credits and permission from Children’s National Hospital Medical Education Department E-Learning Team

Both Google Sites and Articulate 360 contributed to the development of engaging educational cases. Changes were made based on feedback from EM trainees and participants from IPSSW regarding technological pitfalls. Design changes involved collaborating with educational technology specialists and converting to Articulate 360 to address these challenges in three ways. First, clinical images were improved (Figures 6, 7).

**Figure 8:**
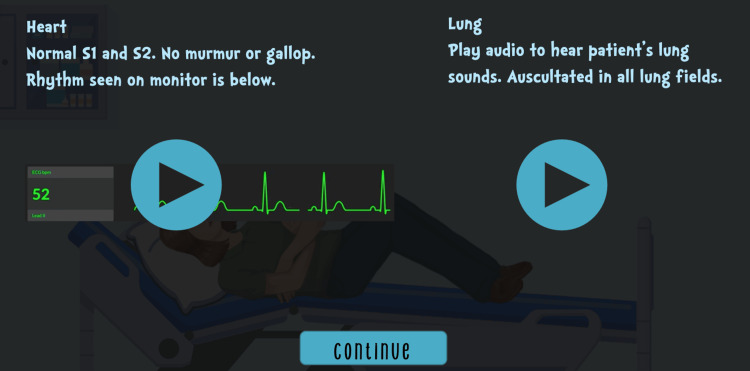
Physical examination findings with audiovisual cues on Articulate 360 Image credits and permission from Children’s National Hospital Medical Education Department E-Learning Team

Audiovisual files were added to the physical examination for the organophosphate case (Figure 8).

Second, pop-up quizzes and puzzles were inserted without needing multiple internet tabs. Third, clues were locked to improve progression through the VER before unlocking the final clue. Identifying the technological benefits of Articulate 360 aided in the development of the iron toxicity case.

Regarding the Articulate 360 case on iron toxicity, respondents wanted to be able to verify answers with feedback, for example, the crossword puzzle and drag-and-drop puzzle. Respondents also requested a progress bar and suggested adding time to the whole scenario each time they used a hint. To address these issues, the VER has been updated to cross out answers on the crossword puzzle and highlight the drag-and-drop puzzle when the answers are in the correct box. A progress bar was added, as well as a hint button, which links to an evidence-based document to help the participants answer questions.

To our knowledge, this is the first study comparing Google Sites and Articulate 360 as platforms for VERs. Previous studies have reported the use of Google, Deck.Toys, and PowerPoint as portals to host VERs; however, they do not compare platforms [2-4,7,13-14]. Our study found that both Google Sites and Articulate 360 achieve the objective of designing an innovative and engaging educational tool. Google Sites is a free resource, but it is more time-consuming. At a minimum, users should have a basic level of technological skill. Google Sites users should learn to navigate the site and practice embedding puzzles. Users may choose to pay for additional features, such as puzzle-developing websites, which have varying costs. The technological challenges encountered on Google Sites were addressed by transitioning to Articulate 360. The time difference in creating the Google Sites and Articulate 360 VERs does not account for each developer’s technological experience. Physicians with intermediate technical skills made the Google Sites VER, whereas the Articulate 360 VERs were developed by the e-learning team with more technological experience and skill.

Regarding production cost/use information at the time of research, Articulate 360 costs $1,399 annually for a team plan license; the rate is $699 with an education discount. In contrast, licenses for a personal plan are $1,099 annually, with an education discount of $549 [9]. Images by the e-learning team were curated from ELB Learning. Licenses cost $1,398 annually (or $117/month) per author per year [15]. In summary, Google Sites is free but takes more time, whereas Articulate 360 combined with ELB Learning subscriptions for images is more expensive and varies based on the plan selected.

This study has several limitations. It focuses on only two platforms, Google Sites and Articulate 360, for VER development, and the participants included EM trainees in India and IPSSW attendees, limiting the generalizability of findings. The facilitator guide for the organophosphate case was tested with participants using Google Sites, whereas Articulate 360 was used for VER-building practice. VER evaluations relied on self-reported surveys, potentially introducing a response bias and preventing assessment of changes in trainee practices or patient outcomes. Future research should consider the benefits and limitations of additional platforms and conduct more comprehensive evaluations of educational outcomes associated with VERs.

## Conclusions

Different portals may be used to develop VERs to gamify education modules. VERs require significant upfront efforts but can be used worldwide. VERs on Google Sites and Articulate 360 are engaging educational tools for distance learning. There are benefits and disadvantages to simulation technologies for Google Sites and Articulate 360. Game-developing platforms such as Articulate 360 can address technological pitfalls. It is essential to get feedback from participants, educators, and developers to identify areas for technological improvements. The simulation community should consider time, funding, and technical requirements when selecting a platform to build a VER.
